# “The most important thing is that those closest to you, understand you”: a nested qualitative study of persons with psychotic disorders’ experiences with family involvement

**DOI:** 10.3389/fpsyt.2023.1138394

**Published:** 2023-05-15

**Authors:** Kristiane M. Hansson, Maria Romøren, Lars Hestmark, Kristin Sverdvik Heiervang, Bente Weimand, Irene Norheim, Reidar Pedersen

**Affiliations:** ^1^Centre for Medical Ethics, Institute of Health and Society, University of Oslo, Oslo, Norway; ^2^Division of Mental Health Services, Akershus University Hospital, Lørenskog, Norway; ^3^Center for Mental Health and Substance Abuse, Faculty of Health and Social Sciences, University of South-Eastern Norway, Drammen, Norway; ^4^Division of Mental Health and Addiction, Vestre Viken Hospital Trust, Drammen, Norway

**Keywords:** schizophrenia, severe mental illness, family interventions, family psychoeducation, patient experiences, qualitative research, implementation, process evaluation

## Abstract

**Introduction:**

Family interventions constitute effective treatment for persons with psychotic disorders. However, the active ingredients and beneficial processes of these interventions are insufficiently examined, and qualitative explorations of patients` experiences are lacking. This study was nested in a cluster randomised trial that implemented national guidelines on family involvement in Norwegian community mental health centres, including family psychoeducation and basic family involvement and support. The aim of this sub-study was to explore how patients with psychotic disorders experience systematic family involvement, and its significance.

**Methods:**

We conducted semi-structured, individual interviews with 13 persons with a psychotic disorder after systematic family involvement. The participants were recruited through purposive sampling. Qualitative content analysis guided the analysis.

**Results:**

Participants reported overall positive experiences with systematic family involvement. It was significant that the relatives increasingly understood more about psychosis and their situation, while they themselves also gained more insight into the relatives` situation. The participants emphasised the need to enable both patients and relatives to safely share experiences in a containing space, led by professionals. Shared understanding and awareness of each other’s situation further improved communication, coping with the illness, reduced stress, and stimulated a more caring family environment. The therapist seemed crucial to facilitate these beneficial communication processes, and also to provide continuous support to the relatives. Reported challenges included that the participants felt vulnerable in the initial phase, a need for tailored approaches, and too late start-up.

**Conclusion:**

Findings from this study suggest that persons with psychotic disorders may benefit greatly from participating in systematic family involvement. This study also gives new insight into possible mediators of positive outcomes both for the patients and the relatives. Systematic family involvement should be implemented a standard approach in the early phase of the disease, using a step-wise and tailored process.

## 1. Introduction

Family involvement interventions, together with pharmacological treatment and individual therapy are the key treatments recommended in clinical guidelines for the assessment, treatment, and follow-up of persons with psychotic disorders (1). Psychotic disorders (2) are severe mental disorders that may highly impact the life development of the affected individuals and their families (3). To experience psychosis has been described as “a state of confusion, where mental and emotional chaos has arisen, and where the most important characteristic is a reduced ability or inability to distinguish between oneself and the reality around oneself” (4). Although the symptoms, experiences, and severity of psychosis vary greatly among individuals (5), many patients may experience severe, enduring symptoms, debilitated psychosocial functioning, and reduced quality of life (6). Moreover, increased care burden for the relatives of individuals with psychotic disorders is reported frequently, such as relatives facing significant stressors, including not receiving timely information and support when it is needed (7–10). Furthermore, the psychotic disorders’ impact on the family dynamics is widely recognised in terms of dysfunctional communication patterns, high levels of expressed emotion (EE), and family disruptions (6, 7, 11). Psychotic disorders also have a vast socio-economic impact (4), imposing large costs on the health and welfare systems (12).

Family involvement interventions, such as family psychoeducation (FPE) (6) which is further described below, are effective and highly recommended types of treatment (1, 13–15). Research on such interventions has persistently demonstrated significant and robust outcomes for patients and relatives (6, 16–20), and the efficacy of family interventions in reducing relapse rates is particularly well documented (16, 19, 21, 22). A core function of FPE, and other similar interventions, is to alleviate the devastating processes that may arise within the family environment, due to psychosis. These processes are well described in the model of reciprocal causation (6), which has increasingly recognised the impact of stressors as mediating factors of exacerbations. In the context of psychosis, reducing patient and relative stressors is therefore of great importance.

However, there are severe obstacles and knowledge gaps hindering the use of systematic family involvement in mental health care (23, 24). As such, interventions can be considered complex interventions (25), and implementation is by nature associated with multilevel barriers (23, 26, 27). Furthermore, the mechanisms by which family interventions can stimulate positive outcomes are far from sufficiently investigated and understood (17, 19, 28, 29). When evaluating complex healthcare interventions, the Medical Research Council’s guidance (30) recommends that outcome evaluation should be complemented by process evaluation, for example to evaluate the quality and acceptability of implementation, and to explore possible causal mechanisms (31). Furthermore, attempts to scale up and optimise family involvement practices should be based on appropriate evidence. This includes knowledge that is informed by all stakeholders (28), as insights into first-hand experiences with systematic family involvement are crucial to deliver high quality family interventions in real-world clinical settings, and to get more knowledge about possible factors that may contribute to positive and negative outcomes. Thus, to evaluate complex interventions—like systematic family involvement—we need both qualitative and quantitative research (30).

However, only a few qualitative studies have explored FPE and similar interventions in depth from the perspective of patients with psychotic disorders (32–36). To learn more about the patients’ experiences with both basic family involvement (BFIS, further described below) and FPE, and to explore possible dynamics and mediating factors, we performed a qualitative study as part of a comprehensive evaluation of a randomised trial. To our knowledge, this is the first study based on interviews with this patient group about their experiences of receiving a combination of single-family FPE groups and BFIS. The study was guided by the following research question: How do patients with psychotic disorders experience systematic family involvement, and what significance does this family involvement have?

## 2. Materials and methods

This article complies with the “Standards for Reporting Qualitative Research (SRQR) checklist” (37) (Supplementary file 1).

### 2.1. Setting, design and intervention

This article is based on a qualitative sub-study of the Implementation of Family Involvement for persons with Psychotic disorders—IFIP-study (24, 38), a large cluster randomised study on implementation of the Norwegian national guidelines on family involvement for persons with psychotic disorders that was conducted in Norwegian community mental health centres (CMHCs) from 2017 to 2022. Fifteen clinical units from 12 CMHCs in South-Eastern Norway participated in the study. Participating units differed greatly in terms of their patient population, service type, and level of family involvement at baseline (24). The IFIP-study was established to improve health services and the health of individuals with psychotic disorders and their relatives through implementing evidence-based national recommendations on family involvement. Based on the national guidelines (1, 39, 40), the project group developed the IFIP-interventions (Table 1) which is thoroughly described in the protocol article (38) and the fidelity outcomes article (41).

**Table 1:** Implementation of Family Involvement for persons with Psychotic disorders (IFIP) intervention.

IFIP: Implementation interventions
*Family coordinator (FC):* A local health professional appointed to help implement, coordinate, and sustain the practice. Should be appointed immediately after randomisation.
*Implementation team (IT):* A local team of 4–5 persons, including the FC, service user- and/or relative representatives, and the unit leader, to plan and supervise the implementation process with assistance from project researchers. Should be constituted immediately after randomisation.
*Training and supervision:* • Clinical training: 4 days interactive course and monthly clinical supervision for 1 year. • Supervision and training days with feedback on fidelity results and teaching sessions.
*Toolkit and shared resources:* Guidelines, FPE manual, BFIS conversation guide, lectures, fidelity instruments, examples of procedures, documentation templates, and information leaflets, barriers- and facilitators guide, web resources, and films.
*Fidelity measurements:* Regular measurements of the adherence to the guidelines, using fidelity scales, with tailored on-site feedback and implementation supervision of IT and FC (formative evaluation).
IFIP: Clinical interventions
*Basic Family Involvement and Support (BFIS):* • Written information about severe mental illness, treatment, family involvement, health services, available support measures such as seminars and peer support, and web resources, for both service users and relatives. • At least three conversations (C) about FI: C1: Service user and clinician. C2: Relative(s) and clinician. C3: Service user, clinician, and relative(s). • Crisis/coping plan to document warning signals, strategies for preventing illness deterioration and relapse, the service user’s wishes regarding treatment, and emergency services and contact numbers. • Psychoeducative seminars for relatives about severe mental illness, treatment and rehabilitation, and the carer role.
*Family psychoeducation (FPE):* • Engagement and alliance sessions (similar to the three conversations of BFIS). • Identifying warning signals, developing a crisis/coping plan and genogram, discuss goals of treatment. • Psychoeducation: Tailored information about illness, treatment, rehabilitation, relapse prevention, etc. • Communication skills and exercises to promote constructive communication and reduce criticism. • Problem-solving sessions: A structured approach to identify issues and work on solutions together.

The clinical interventions of the IFIP-study consist of Basic Family Involvement and Support (BFIS) and FPE in single-family groups. BFIS refers to three separate conversations about family involvement: one with the patient, one with the relative(s), and then joint conversation(s). This is in addition to written information about family involvement and support, seminars for relatives, and a crisis/coping plan. FPE is an evidence and manual-based model (6, 42, 43) that provides psychoeducation about the disorder, emotional support, means to improve stress coping, problem solving, communication skills, and crisis management (44). Ideally, an FPE course should be 4–9 months in duration and start with separate alliance sessions with patients and relative(s), followed by joint sessions. Among our study participants, 9 out of 13 had participated in FPE alliance and joint sessions at the time of the interviews, and the various participants may have participated in both BFIS and FPE, or only one of them. At the initial phase of implementation, all clinicians, leaders, and resource personnel were invited to attend a four day FPE training programme (45), followed by regular supervision throughout the intervention period.

In the IFIP-study, doing other types of family involvement before or during the trial was not an exclusion criteria. Thus, some of the units also did other types of family involvement than FPE and BFIS, such as other types of systematic family interventions or more unsystematic forms of conversations, for example when the next of kin called the service units requesting information on how to support the patient.

### 2.2. Inclusion and participants

We used a purposive sampling strategy (46) to ensure explorations of patients’ experiences with systematic family involvement. Inclusion criteria included: psychotic disorder or currently undergoing psychiatric evaluation to determine whether the patient have a psychotic disorder, 18 years of age or older, capacity to consent, and exposure to BFIS and/or FPE in the intervention period. All participating units used standardised and often several measures to diagnose psychosis, such as Positive and Negative Syndrome Scale (PANSS) (47), Structured Clinical Interview for DSM Disorders (SCID) (48), and specialist consensus. Exclusion criteria included: not being competent to consent, not having any relatives, being a forensic patient, or having an increased risk of violence.

The recruitment process involved several contributors at the CMHCs. Initially, the unit leader, family coordinator, or research coordinator received information about the recruitment procedures. This information was further provided to the respective clinicians who were encouraged to assess eligible patients, provide them with proper information, e.g. their right to withdraw from the study without reason at any point, and obtain consent to participate in the study. The clinicians were asked to include patients with both short- and long-term illness, patients with both positive and negative experiences with systematic family involvement, and a wide distribution in age and gender. The participants did not receive compensation for participating in the interviews.

### 2.3. Data collection

We conducted 13 individual interviews with patients, all with an established psychotic disorder, during spring 2020. The interviews lasted approximately one hour and were performed by MR and KMH, who both have extensive experience with conducting qualitative research on vulnerable groups. Physical attendance was not possible due to the Coronavirus pandemic, thus six participants were interviewed by phone, and seven by a digital conference platform (Zoom) (49). The interviews were guided by a semi-structured interview guide (Supplementary file 2). To ensure applicability, the guide was developed through input from several of the researchers in the IFIP group, and further piloted with a representative from the Norwegian user organisation Mental Health. Prior to each interview, the researcher in charge contacted the patient to schedule the interview and clarify whether the patient preferred to participate via telephone or Zoom.

Initially, the participants were asked to identify significant persons in their everyday life and describe how these had been involved in the treatment at the CMHC. We further explored their views of the benefits and significance of systematic family involvement to themselves and to their family, but also challenges and potential disadvantages. The participants were also encouraged to share their views on how their therapist and relatives could facilitate positive experiences with family involvement. When utterances particularly relevant to the research question occurred, we asked follow-up questions to stimulate further elaboration. The interviews were audio-recorded on external dictaphones, transcribed verbatim and immediately transferred to the University of Oslo’s secure database (In Norwegian: “Tjenester for Sensitive Data”).

### 2.4. Analysis

Immediately after each interview, a brief report was written by the interviewer (researcher) to summarise immediate impressions and recurring themes. This initial process of analysis also stimulated researcher reflexivity concerning the interview performance, provided co-authors with initial data familiarisation, and formed the basis for discussions among KMH, RP, MR, BW, LH and KSH on preliminary findings. The first author applied qualitative content analysis (50, 51) to explore the interviews and NVivo computer software package 12 was used to structure the analysis. The transcripts (unit of analysis) were read through several times to obtain a sense of the whole (50). To identify various relationships and themes within data, a non-linear process of de-contextualisation and re-contextualisation further took place, which simultaneously involved abstraction and interpretation (51). The process of de-contextualising involved separating data from their context to uncover all participants’ statements about the phenomenon in question. The material was descriptively coded by dividing it into separate meaning units and labelling each unit with a word or phrase (manifest content with low degree of interpretation and abstraction), for example “learning about psychosis.” The process of selecting text excerpts and coding resulted in some comprehensive meaning units. This was deemed necessary to avoid unfortunate fragmentation of descriptions of individual’s experiences with a complex phenomenon (50, 51). If a solitary code seemed to fit in more than one category/sub-theme, the code was placed into each (50). Re-contextualisation constituted the interpretation of data and refers to combining the various utterances into new patterns and relationships, allowing a deeper understanding of the phenomenon under investigation. Data were subjected to further grouping of codes into higher-level categories and themes (latent content, increasing degree of interpretation and abstraction). RP, MR, LH, and KH reviewed the final analysis by discussing the content and levels of abstraction in the thematic map until agreement on the final categorisation was obtained. This collaborative process resulted in the final division of the material into two unifying “red threads” (52)—the overarching themes. Pseudonyms are used to obtain anonymity. Each patient participant got his or her own pseudonym in the analyses, to make it easy to assess whether quotations are from the same or different interviews.

### 2.5. Research ethics

The study has been approved by the Norwegian regional committee for medical and health research ethics Southeast with registration number 2018/128, and by the local data protection officers at the participating units and at the University of Oslo, to ensure that the study was carried out in accordance with relevant regulations and guidelines. All participants gave a written and informed consent, and confidentiality and privacy has been ensured.

This study included vulnerable participants—that is patients with psychotic disorders. This was considered well-justified since this patient group has been relatively neglected in qualitative research on systematic family involvement.

However, particular ethical consciousness towards the study participants was thus required. For example, to make the interview situation less stressful, and to ensure that the participants fully understood which topics would be addressed during the interview, they were provided with the interview guide in advance. During the interviews, we strived to make the participants comfortable by being conscious of our appearance as researchers, meeting the participants with active listening, empathy, and sincere interest, and allowing for individual adaptations—such as providing short breaks where needed or the opportunity to turn off the screen during the interviews. At the end, we asked the participants how it had been to participate and whether they needed extra follow-up from their therapist in the aftermath. Several expressed that contributing to the research like this were experienced as meaningful.

## 3. Results

Thirteen patients with an established psychotic disorder was included in the study. Table 2 presents background characteristics of the participants.

**Table 2:** Participant characteristics (*n* = 13).

Female gender, *n* (%)	8 (62)
Age, *mean (range)*	37.3 (20–60)
Ethnicity, *n* (%)	
Norwegian	11 (85)
Other	2 (15)
Years since time of diagnosis, *n* (%)	
0–5 years	7 (54)
10–20 years	3 (23)
> 20 years	3 (23)
Next-of-kin^*^ participating in family involvement, *n* (%)	
Spouse/partner	5 (38)
Parents	9 (69)
Siblings	5 (38)
Children	1 (8)
Other	2 (15)

* Each patient had one or more next-of-kin participating.

**Figure 1:**
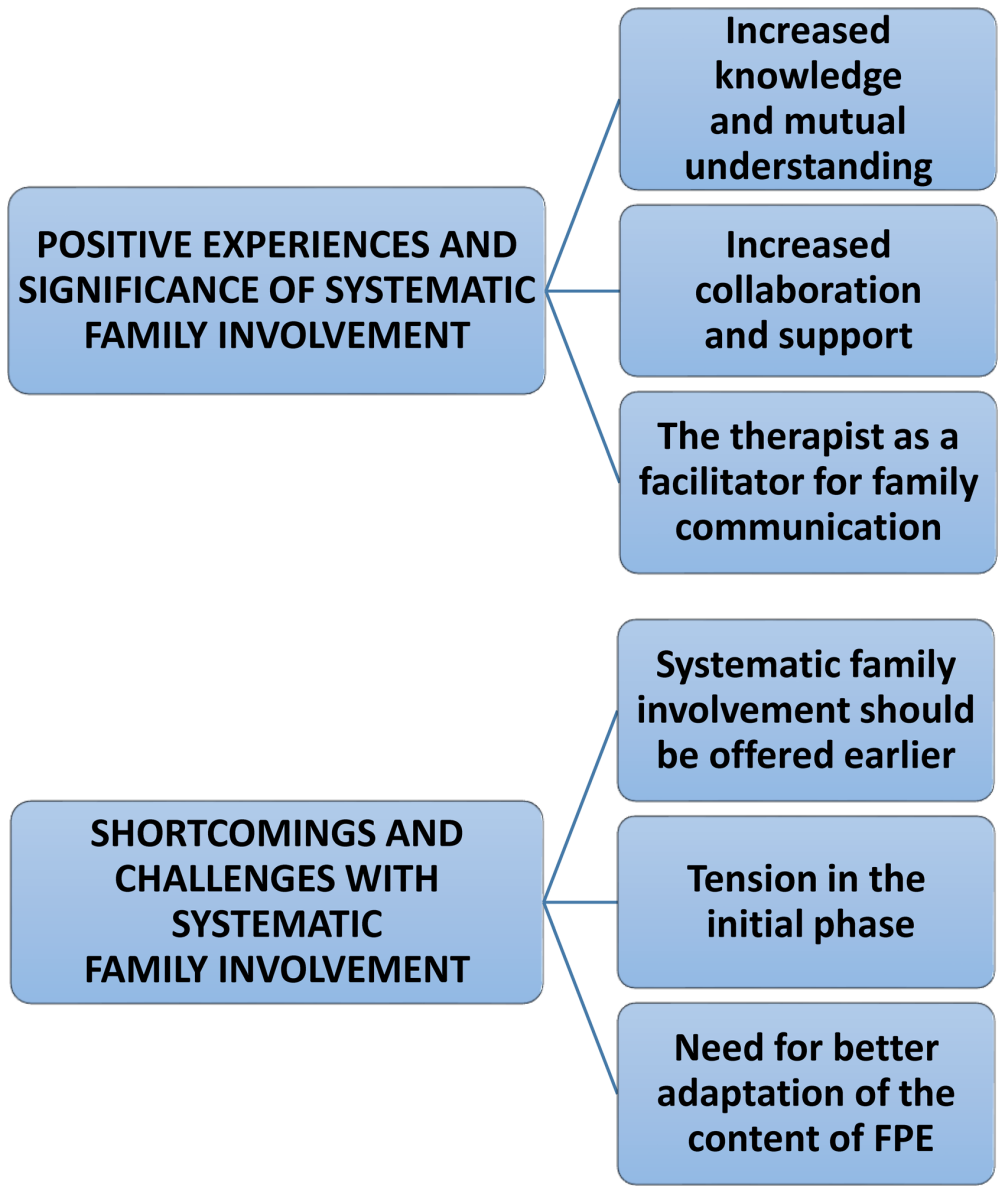
Thematic map of patient experiences and significance of systematic family involvement.

Analysis resulted in two main themes: (1) Positive experiences with and significance of systematic family involvement, and (2) Shortcomings and challenges with systematic family involvement (Figure 1).

The participants described more general experiences and consequences of living with mental illness from before attending the study. Suffering from a psychotic disorder had negatively affected several of the participants throughout life in terms of experiencing hostility, conflicting understandings, loneliness, and difficulties in expressing their inner state or situation to those around them: “I did not feel like anyone understood me, talked to me or listened to me…,” Susannah said. Cathrine detailed how unwanted patterns had arisen within her family: “If you have nothing to defend yourself with, having a hard time and in some way are being pushed into a corner and being faced with accusations… This is the kind of experience I have, but it does not mean that this is what they [the parents] want. The situation gets very tense, everyone is defending themselves, and the family dynamic becomes something that no one wants to deal with. Then we just stand there…”.

Participants were also concerned about the burden the disease had caused their relatives, such as feelings of guilt, fear, and despair and, in some cases, deterioration of health: **“**My illness became such a strain on my partner that he also became ill,” said Susannah. Axel, for his part, expressed how his brother was paying the price for the lack of family involvement: **“**The biggest problem is probably that my brother did not get involved … He is left with the biggest problem of us all really, because he has received the least information … thus from the beginning he thought that it was he who had been a bad brother.”

Most often the participants did not distinguish between BFIS and FPE, thus we mainly refer to the term “family involvement” as comprising both. When it was obvious that the participants were referring to FPE, this is specified.

### 3.1. Positive experiences and significance of systematic family involvement

Participants across the interviews reported overall positive experiences with the systematic family involvement they had been offered, despite there being large variability among them in terms of their experiences with such interventions, how long they had been ill, who their relatives were, as well as their age and gender.

#### 3.1.1. Increased knowledge and mutual understanding

This theme refers to statements about how family involvement contributed to increase the patients and their relatives overall knowledge and understanding. Psychoeducation was perceived vital to gain a more thorough understanding of psychosis. Hannah noted that learning about psychosis had strengthened her cohabitant in the carer role: “I think my partner found it very useful. If I experience stress … how to avoid it, other causes of psychosis, prevent it a little.” Besides learning about psychosis, most participants reported a high value of getting insight into each other’s situations and views. Caroline felt that participating in FPE had made her family less critical: “If there is something that is… overwhelming, or a little difficult, they are beginning to understand more that this is not what I do to be difficult but that I have a need to do the things I do.” John felt a renewed relation to his father: “The most important positive change that happened was that my father finally understood that there was something wrong then … that I have not been well for a long time … he got it like a punch in the face, as he said himself … and he has changed for the better after that, I think.” The researcher asked him what had changed: “He does not push me so much anymore … has started to show interest … he is more into the conversation when I tell him about things I like, and he listens better.”

The increased understanding was not only about the relatives understanding of the patient. The participants also reported an expansion in their awareness of their family members situation: “They have been very worried … and it has put weight on them, I have not realised how tough it has been for them, right … because I have been so preoccupied with myself, my problems. I have not been able to see the problems they have had along the way. Which are not just my problems … but everything around. That they had a tough life that wasn’t really about my story anymore, now it was about their story,” Axel said. Emily described how gaining knowledge and understanding made her regard her family more positively: “I think I have gained more trust in them, and perhaps have … gained more patience … I still have moments where I do not trust almost anyone, but now I see more what their real purpose is—that they want to support me.”

Experiencing a relational shift was a recurring theme. Cathrine, who had been previously receiving critique from her surroundings, said: “They treat me differently … My brother said, for example [before]: “You can smile more, cannot you? You almost never smile. You can be a little happier.” This was also the case for Emily and her aunt, who communicated better when mutual understanding increased: “My aunt has in a way learned more about what I really need and how things are for me … she did not have much insight into things before … she can perhaps be a bit like ‘cannot you just do this and that, or just stop taking your medication, or stop putting on weight?”—maybe this has changed … she has more patience with me, she understands me better. I also feel that, in a way, I have gained more trust in her.”

In Axel and his mother’s case, understanding each other better was not about coming to a full agreement but rather finally being able to communicate, recognise, and accept that they carried different experiences and perceptions of how life with the illness had been. Axel said: “My mother said that “based on everything that has happened, you have experienced something and I have experienced something, but we have not experienced it together.” And when acknowledging this, she has actually got the answers she needs. Because then she can see that I see it differently … and I can understand that she sees it differently. This is something of the most important I have gained from FPE.”

#### 3.1.2. Increased collaboration and support

This theme describes accounts concerning enhanced support and collaboration among the participants, relatives, and family system.

##### 3.1.2.1. Perceived support

In the participants’ view, increased knowledge and understanding enabled their relatives to provide better support: “The best thing I have gotten out of it is more understanding in the family. They can help me adapt…help me get through tough days…” John said, while Anna expressed the importance of having her husband involved: “It has been very nice to have someone next to me who sees my problem and solves it—and grows together with me.” Several accounts concerned how the patient felt they were met with greater interest and warmth from their relatives. Participating in FPE promoted emotional support: “They understand more in the family. You are not so alone with your problems” (Rita), “It means a lot. The support I get all the time and understanding and help when I need it” (Anna), while Emily experienced that her social function had improved: “I can be safer/more secure in the social contexts because those closest to me are more involved in how I feel and know more about what I am going through.”

All in all, the interviews gave the impression that when the services were involved and supported their family, quality of life ameliorated. Emily, for instance, talked about how systematic family involvement had given her an increased sense of predictability and coherence in life: “I feel that I have gained a little more security in those relationships…it is more predictable…if there are many things I am thinking about, I know that I can bring this up at such a meeting. So I feel that my life has somehow become more complete, in some way, after we have had that collaboration.”

##### 3.1.2.2. Improved problem solving in everyday life

Several participants noted that the problem-solving sessions stimulated the family collaboration. The families increasingly dealt with their problems—individually and within the family—through a more solution-oriented approach, and began solving problems together and working towards shared goals—which mitigated their everyday life. Magnus said that “the problem solving was good since, yes, we have to organise the everyday life together … so then it was certainly useful to plan on two fronts,” while Susannah appreciated attending the problem solving sessions regularly: “Problem solving every 14 days was really great … Because then we could work on a new problem solution in everyday life, then we could talk about problems, things we wanted to fix, then we made a little plan, and then we were testing it until the next session.” Susannah was also relieved that the FPE-sessions were problem-focused rather than person-focused: “I had imagined it would be very uncomfortable…very personal. Rather, we addressed a problem that is bothering me at the moment, and we discussed what everyone could do to make it better. It was a very pleasant way of doing it, focusing on a specific problem rather than what I had envisioned.” Anna expressed how communication improved through being together: “It has been very nice to sit together and talk about everyday life … they can hear about my difficulties and about my anxiety—try to find help for me … The communication between me, my husband and the therapist becomes good when we resolve our thoughts together.”

##### 3.1.2.3. Help to prevent a relapse

The knowledge and skills gained through FPE were linked explicitly to relapse prevention: “The symptoms have been stopped from…becoming psychosis. By using the things we have set up for the family collaboration … warning signals indicating things are getting difficult and …what I have to do to not being ill,” Rita noted. Magnus explained the value of having a crisis plan when his condition deteriorated: “It’s quite difficult for me to take action when I get sick, it’s somehow much better when someone else notices it … the one who takes action then is primarily my mother … then she calls the CMHC and gets an admission.”

##### 3.1.2.4. The relatives are taken care of

Participants also shared how they considered their relatives to benefit from family involvement for their own sake. Emily valued how the services were supporting her family: “It is very nice that the relatives can get involved (in the treatment), and at the same time they can be “protected” in a way, by the health care services.” Alliance sessions were described as highly valuable for enabling the relatives to speaking openly about issues that they were reluctant to disclose in the presence of the patient. Susannah said of her husband: “he is able to ask questions that he finds uncomfortable asking me. And finally he can get some decent answers.” Christian, for his part, reflected on how his mother had benefitted from the conversations with his therapist: “The first time, my mother was so nervous. But then she was so satisfied … it helped her to talk a little … There is probably a lot she is ashamed of. You know, it’s no medallion to have a drug addict son.”

Some of the participants also appreciated that their therapist had provided their relatives with an open line to the services. Firstly, the patients recognised how such continuous support reduced their family members’ stress and feelings of carrying the burden alone. Secondly, they also considered it to have a positive impact on themselves and the family dynamics. A recent episode in Rita’s life was used to exemplify how such an open line of communication could potentially prevent a deterioration of the family climate: “My husband and mother have the therapist’s mobile number. Lately they have been worried about my food intake. Then I think it is better that they call therapist rather than them taking out their frustration on me.”

#### 3.1.3. The therapist as a facilitator of family communication

This theme concerns the role of the therapist during systematic family involvement, who seemed to play an important function in facilitating the enhanced knowledge, understanding, collaboration, and support outlined in the former themes.

##### 3.1.3.1. Facilitator of patient-relative communication

The participants reported that, in their opinion, the therapist was critical in supporting them in their communication with the relatives. Christian, for example, expressed how he expected that having the therapist by his side would help him to express himself to his mother in a forthcoming session: “I get a little scared when I think about me, the therapist and my mum shall sit and talk together, because I have not done that for a long time … I’m not lying that I’ve been high, but I do not want to worry her … but with the therapist there … He is quite stable, so I think I will be able to convey it well with his help.” Caroline, for her part, was relieved that the therapist had conveyed information about her on her behalf: “I find it difficult to talk to my parents about how I feel. I have not been able to do it myself, so I think it was very nice for them to hear such a calm version of how things are, and to be able to ask the questions they had.” To Monica, relying on professional authority when communicating with her mother and children had been imperative: “I feel a great relief that they are family therapists because then I do not have to try to get them to understand. It’s coming from someone who knows their subject.”

##### 3.1.3.2. Creator of a safe and containing space

FPE components like psychoeducation, emotional support, and communication rules facilitated by a supporting therapist laid the groundwork for an increased openness regarding mental health within the family. While previous issues with stigma, shame, and difficulties with talking about mental illness were repeatedly addressed by participants, FPE sessions were described as a means of providing dedicated time and a confined space for dealing with sensitive topics which was not possible in their everyday life alone with their relatives. Being able to talk more openly about sensitive issues and each individual’s struggles seemed to dampen the emotional pressure in some of the families: “There has been less despair about the whole thing,” said Martin.

### 3.2. Shortcomings and challenges related to systematic family involvement

#### 3.2.1. Systematic family involvement should be offered earlier

The interviews unveiled how some participants during times of severe relapse felt an unmet need for help to involve and interact with their family. They pointed at systematic family involvement as a measure they should have received earlier in life—for their own and/or their relatives sake. For example, Monica said that “My family should have been involved when I first got sick. When I started at the psychologist’s, when it all started, the child protection services and everything…” Susannah noted that her husband had benefitted greatly from the alliance sessions, but “he wished it could come a little earlier.” While Susannah was offered one-to-one conversations at initial hospitalisation, her husband had to wait for 6 months before receiving any information from the unit. The problem was that he needed these conversations the most at the beginning, information about the prognosis and what would happen next, as soon as his wife had become seriously ill.

Two participants shared their views and advice to future patients not to exclude the family when they get ill. Christian for example was very concerned with his own troubled story, and wished for others to avoid the mistakes that he and the health and social services had formerly made: “Try to reach out to your family before it goes to hell. If I had managed that, I would have been sober by now. I did not make it, that’s why I’m saying it … Just try …they love you.”

#### 3.2.2. Tension in the initial phase

A few participants felt particularly vulnerable and uncertain when being asked for consent to involve their family and before attending the joint sessions: **“**It’s not that I do not want to, but I’ve been afraid. Until now.,” Christian explained, while Martin pointed out that patients can be reluctant to consent because of a felt need to protect their relatives: “I do not want to be a burden.” Caroline found the waiting time before the joint sessions very challenging: “I imagined that it was going to be me who had to speak, or that it was going to be uncomfortable. Such meetings [FPE-sessions] are often set up a long time in advance, so there were many weeks of uncertainty. I lived in my parents` house then too—yes, I think it was a bit difficult. Because at the same time they met with my therapist [alliance sessions]. I think it was uncomfortable that they met each other while I was just waiting, in a way.”

However, both Christian and Caroline learned that their fears were unfounded when they finally started with FPE. Caroline added that being reassured during the alliance sessions that her views on information disclosure were taken into account was helpful: “One thing I really liked was that I got to decide what the therapist was going to say to my parents.” When asked what action they needed from the therapist to make the initial phase more comfortable, Caroline responded: “Just make it very clear that it is not as unpleasant as one might imagine, then. It is rather just that we look at the problems together …,” while Christian was content with the therapist’s patient approach: “He has done it completely right, because he has taken it carefully. He understands “that the things I struggle with are quite bad, so he has moved slowly forward, talked a little about what he and my mother have talked about—and maybe in the future we will have a joint meeting.”

#### 3.2.3. Need for better adaptation of the content of FPE

Some statements revealed that the FPE content was not sufficiently adapted to the individual family and their specific situation. John felt that far too much time was spent on problem solving: “It’s been interesting, but it has also been very … much of the same every time [laughs]. In the end it was just like … ‘now we’ll have to come up with something’.” Rita experienced conflicts, but emphasised that she did not think the therapist could have done anything differently:” Twice I did not accept my dad’s suggestions. The second time he was offended and wanted to quit the group … but I spoke to the therapist on the phone afterwards, and was reassured that it was not me being the problem.”

Susannah had some suggestions for improvements. Firstly, she wished there had been more focus on her husband in the joint sessions: “There has been a lot, a lot of focus on me and ‘my things’. Perhaps [there should have been] even more focus on my partner.” Secondly, she felt that separate conversations with the therapist alone where she could express herself in private had come at the expense of the joint sessions.

## 4. Discussion

Through patients’ accounts, we found that enhanced knowledge and understanding became key mediators of collaboration and support. It was imperative to the patients that their closest ones had knowledge about their illness and understood their situation, strains, and needs. While a lack of understanding seemed to promote stress, conflicting communication, and worsening of disease, the new understanding and awareness of each other’s situation improved communication, reduced stress, stimulated a more supporting family environment, and improved the overall coping with the illness. The therapist functioned as a facilitator of patient-relative communication, creator of a safe, containing space, and as a source of continuous support to the relatives. It also seemed that the therapist was crucial during the initial phase of FPE, to reduce tension and stress and patiently pave the way for the patient into systematic family involvement. Shortcomings related to systematic family involvement were that the participants felt vulnerable and uncertain in the initial phase, a need for better adaptation of the content of FPE, and that systematic family involvement should be offered earlier.

### 4.1. How systematic family involvement improved the family dynamics

**Figure 2:**
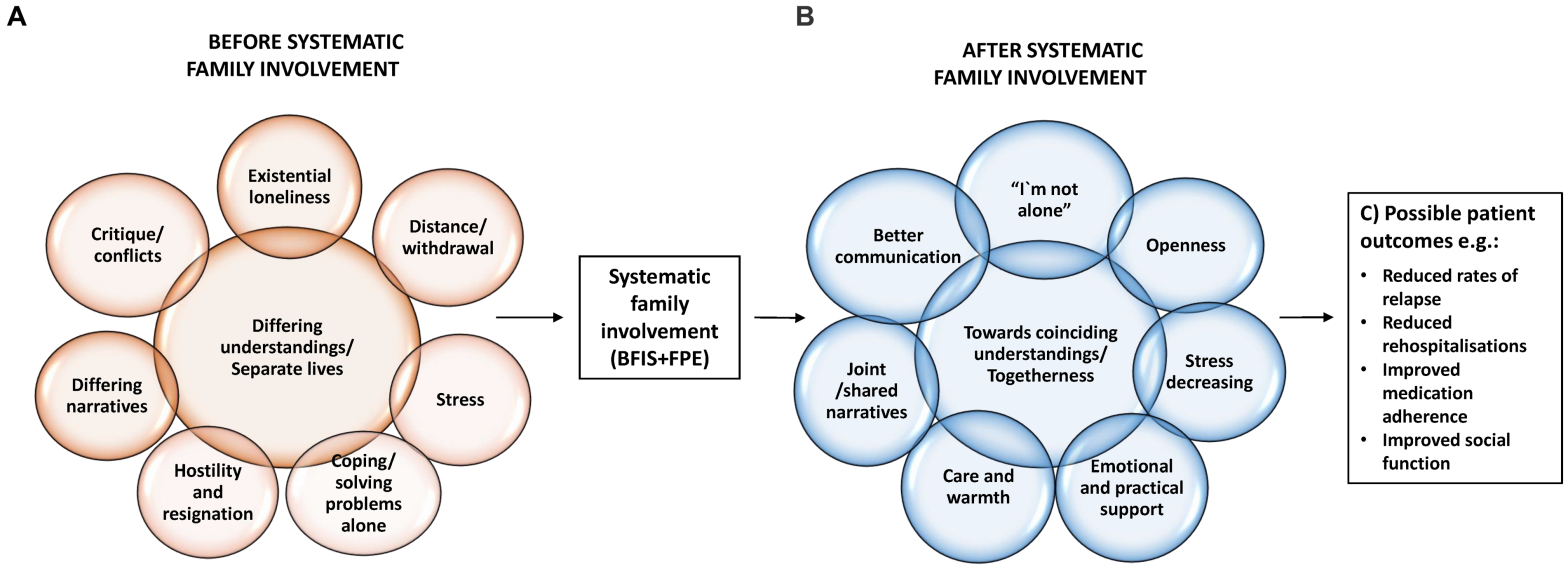
**(A–C)** Systematic family involvement: key processes and possible patient outcomes.

Being severely mentally ill had had a devastating impact on the patients’ life and relationships. Several patients wished that they had received help with how to interact with their family at an earlier time to avoid the suffering they had undergone (Figure 2A). The patients’ narratives demonstrated that participating in family involvement contributed to a positive change within the family system (Figure 2B). In the following section, we discuss the potential mechanisms of impact that led to such a relational turnaround:

#### 4.1.1. Access to knowledge and guidance to understand psychosis

Psychotic disorders are complex biopsychosocial illnesses (35), thus understanding how they develop and potentially influence the afflicted individuals and their surroundings is challenging without help from professionals. The psychoeducational component of FPE provided the patients with access to knowledge about the illness, the diathesis-stress model, treatment options, and prognosis. This was experienced as helpful for understanding themselves, coping with life with severe illness, and creating hope for the future, which in line with Nilsen et al. (33).

Furthermore, psychoeducation worked as a supportive means to convey complex medical information to the relatives. We cannot expect relatives to deal with the devastating consequences of psychosis and provide good care without adequate knowledge and coping skills. However, the patients in this study voiced how they struggled with expressing their challenges and needs, and how poor communication on several occasions had deprived their social life. To reduce loneliness, be understood and supported, the patients needed help to communicate effectively with their peers, which was accomplished through FPE. Before attending FPE, conflicts and critique had arisen in several of the families due to divergent perceptions and understandings. When knowledge about psychosis increased, the relatives seemed to adjust their expectations, both of the patient and of the treatment. This demonstrates the significance of providing psychoeducation to relatives at an early phase of the illness trajectory. Only when relatives’ expectations of their seriously ill family member are realistic it is possible to work coherently towards common goals.

#### 4.1.2. Experiences of being acknowledged and understood

Another core significance of FPE was that the patients were enabled to express themselves in a safe and contained room (35), for some it was the first time since they got ill. Such communication facilitated healing experiences of being met, acknowledged, and understood, both by the therapist and significant persons in their everyday life. This is in line with previous studies identifying “common therapeutic factors” as mediators of the positive outcomes of systematic family involvement identified in the literature (53).

An important point is that, in contrast to individual therapy, the FPE model also provided their relatives access to therapeutic experiences, which was highly valued across the interviews. Research has shown that relatives may have an intense need to share their narratives about caring for an individual with severe mental illness (53). Our findings highlight that it is significant also for the patients that their relatives are given the opportunity to speak out and be acknowledged by the therapist. During alliance sessions their family members could disclose the fear, doubt and grief that often accompanies the caring role—again, for some it was the first such opportunity since the patient’s diagnosis. These healing dynamics can be understood as “acknowledging communication,” a milieu-therapeutic approach commonly used in mental health care. “Mutuality” is a core concept of acknowledging communication and refers to this inter-subjective sharing of feelings and beliefs, performed in a respectful way (54). The “open line” to the services which some relatives had been offered, also contributed to coping with the illness. Especially in times of illness deterioration, having the therapist as a “lifeline” was imperative to manage providing care while also caring for themselves in demanding situations. These findings support the studies showing that relatives’ expressed emotions are identified as robust predictors of relapse (29) (Figure 2C).

#### 4.1.3. Taking part in the narratives of their significant others

Attending the FPE joint sessions allowed the patients to be exposed to their relatives narratives. Joint sessions characterised by a supportive climate (for instance, communication rules were said by one participant to create a calm atmosphere) increased the participants understanding of the others’ point of view, experiences, and needs. This led to a crucial change in the relatives` attitude towards the patient: from previously perceiving the patient as “challenging” or with behavioural faults, the family members increasingly attributed challenging behaviour to the illness. Together with psychoeducation, this promoted relatives` reframing, which is a mediating factor of reduced expressed emotions (53). Similar findings has been reported in a qualitative study exploring patients’ and relatives’ perceived benefits after participating in multi-family or single-family FPE-groups following a first episode of psychosis (33).

Strikingly, we also saw this change in attribution among some of the patients. Their beliefs about their relatives’ behaviour had similarly influenced how they related to and interpreted their actions. Listening to the family members concerns enabled them to explore new perspectives of themselves and others, and listen to the family members worries. This finding, showing how systematic family involvement may contribute to balance family relationships, is less elucidated in the scientific literature, which focuses mostly on how family involvement can provide relatives with insight into the patients’ symptoms and situation.

Furthermore, this is relevant factor to assess the pros and cons of single-versus multi-family FPE-groups. Although the sample is small, these finding may indicate that single-family groups may be more suitable to facilitate this reciprocal understanding than multi-family groups, since the single-family approach is more suitable to explore and improve the mutual understanding between the individual patient and his/her family.

The therapist played a key role in facilitating the abovementioned experiences of gaining knowledge of psychosis, being acknowledged, and learning from each other’s narratives. FPE was portrayed by the participants as a safe, contained space (35) in which the families could disclose, discuss, and navigate sensitive topics. The therapist was central to creating these spaces, in strengthening patient-relative communication, providing emotional support, and building trust and alliance within the triad.

### 4.2. Systematic family involvement should start early, focus on the initial phase, and be tailored

Three key findings in this study concerns aspects of timing. First, systematic family involvement should start at the onset of the illness, or as early as possible, to support the afflicted family in a critical phase of their lives. Several of the patients expressed that systematic family involvement had been initiated too late, with negative consequences for them and their family (Figure 2A). This is particularly important in the prodromal phase, in order to prevent young patients in the early stages of their illness from relational disruptions and to facilitate the strengthening of emotional bonds. At this stage, the family is most likely to still be involved, with a potential for building supportive relationships, contrary to what is found to be the case among patients with a long history with severe mental illness (6).

Secondly, particular attention should be given to the initial conversations about systematic family involvement before FPE, and the time span before and during alliance sessions when consent to involve the family has been successfully obtained. The participants seemed especially vulnerable at these moments, expressing how they were burdened by uncertainty, fear, lack of information, and that they dreaded the participation. Similar findings has been reported in a qualitative study exploring patients’ and relatives’ experiences after participating in multi-family or single-family FPE-groups following a first episode of psychosis (33). This is not surprising, taking into account that both the patient and the family may be in state of chaos or crisis in the initial phase of a psychotic disorder. Furthermore, these findings integrate well with previous implementation research, where patient reluctance and lack of consent are identified as core barriers to systematic family involvement for persons with severe mental illness (26). However, they found that their worries and fears were often unfounded; that is when first attending the joint sessions they mostly found it positive. This alludes to yet more important functions of the therapist: providing the patients with thorough information, listening actively to their worries, and demonstrating a sincere intention to involve them as equal partners in decisions regarding information disclosure. Due to the vulnerable situation and the complexity of the intervention, it may be wise to start the most basic type of systematic family involvement (such as BFIS) and to guide the patient through a process with step-wise consent where the more advanced interventions, such as FPE, may be introduced at a later stage. This seems to be in line with the needs and interests expressed by the patients in this study. That is, a step-wise approach may both lower the threshold and increase the likelihood that the patient consent to and benefits also from more advanced family involvement interventions, despite ambivalence in the beginning. Such an approach, is also in line with another IFIP sub study exploring the mental health professionals’ views on barriers and facilitators to family involvement (26).

Finally, it seems to be a need to adapt or tailor the systematic family involvement to the individual patient and family needs. Such adaptation may be easier using a step-wise approach and a single-family group approach if FPE is introduced at after more basic family involvement and support.

### 4.3. Strengths and limitations

Demonstrating causality or to generalise the findings is not possible in qualitative research. However, the findings can provide knowledge of possible mediating factors, and generate hypotheses that can be tested in future studies. Furthermore, the findings may be relevant or transferable to other similar contexts.

A challenge in the IFIP-study is that we do not know for certain what kind of family involvement the participants have been exposed to or how much. This is often the case when evaluating complex interventions in a large scale and real world setting, and when using a design inspired by pragmatic trials, as in the IFIP-study. They may have participated in both BFIS and FPE or only one of them. They may also have been exposed to other types of family involvement. However, the experiences described are most likely related to BFIS and/or FPE, and that entails (for the patients) at least two systematic conversations focusing on family involvement. Furthermore, although many different systematic family interventions exists, it seems like the core components are similar and that as little as two family involvement sessions can give positive effects (21, 55). Thus, the experiences and findings may be relevant also if the interventions or the experiences have been “contaminated” or influenced by other family interventions, and also with few or many family involvement sessions.

A strength of this study is the context and design of how it was performed—in a real world clinical setting, as part of a large cluster randomised study which has succeeded in improving the implementation of systematic family involvement (41). Close collaborations with many researchers in an interdisciplinary research group guided the research, and the authors of this article are well experienced in accommodating vulnerable groups in research and clinical practice. Most likely, this contributed to strengthen the overall quality of findings, and to facilitate trust and openness among the participants and interviewers leading to rich and valid data.

However, the normative position of the researchers can possibly influence data collection and analysis. Nested in a study which aimed at implementing specific interventions, there is a risk of observer bias if the researcher’s expectations or opinions may impact what they perceive or record in a study (56). To increase trustworthiness of findings, this required that the researchers performed ongoing “reflexive objectivity” (57) that is reflecting on how we contributed to the production of knowledge.

An important strength of this study is that it leans on rich first-hand data from a patient group whose voices are seldom heard, and who can find it challenging to participate in research. A further strength concerns the variation in participants in terms of age, gender and length of illness trajectories which gave us rich and varied data. We assume that the design of the recruitment phase most likely contributed to the richness of data as close contact with the patients’ therapists was established by the time of recruitment. Most likely, this facilitated a “tailored” inclusion of patients who were considered well suited to participate meaningfully in the interviews. On the other hand, this implies a risk of sample bias in terms of poorly functioning patients or families not being included, or that the clinicians, whether consciously or not, encouraged the patients that were most satisfied with the intervention. It is also likely that the participants who consented to participate in general had a positive view of family involvement, and that they had mainly positive experiences with the IFIP-intervention. Another potential limitation concerns bias in recall due to the retrospective design of this study.

Furthermore, it would have been a strength if complementary qualitative data from clinicians and relatives, and quantitative data from the study had been analysed, but for practical reasons these data will be published later. Preliminary findings from these two sub-studies, however, indicate strong coherence in findings.

### 4.4. Clinical implications and future research

These findings, although not able to generalise, indicate that systematic family involvement should be routinely offered to patients with psychotic disorders as soon as possible after the onset of illness. However, it is important that the clinicians are responsive to the individual patient and relatives’ situation and adapt the intervention accordingly, and consider to use a step-wise approach. The pitfalls that may arise during the initial phase of family involvement should be given special attention. Therapists should be given relevant training and supervision to be able to facilitate the positive processes described in this study.

To inform the design and application of systematic family involvement interventions in clinical practice for various groups and settings, more qualitative research exploring the active ingredients of systematic family involvement, and how they are exerting their effect, is needed (58). Studies voicing the patient perspective are particularly encouraged. The scope of this study is limited to patients that have participated in systematic family involvement. To optimise future practice and implementation, research should also explore the perceptions of patients who do not consent to systematic family involvement. Research should also investigate how systematic family involvement is delivered and experienced in other settings, such as in in-patient units and the municipalities.

## 5. Conclusion

The patients in this study reported overall positive experiences with systematic family involvement during psychotic illness and reported immediate and long-term impacts for themselves, their relatives, and the family environment. Engaging with their relatives, with help from professionals, led to a series of meaningful changes related to family interaction. Common therapeutic factors, education about the illness, and problem solving facilitated increased knowledge of psychosis and mutual understandings of each other’s situation and experiences. These new insights further stimulated a more collaborative and supportive family environment that promoted better overall coping with the psychotic disorder and its ripple effects on the family system and everyday life. The therapist was critical in promoting these processes as a facilitator of patient-relative communication, a creator of a safe, contained space, and continuous support for the relatives. “Helping the helpers” was described as imperative to prevent relapse and promote health and wellbeing among both patients and relatives. The findings indicate that it is important to start with systematic family involvement early after the onset of a psychotic disorder, to pay special attention to the initial phase of family involvement, use a step-wise approach, and ensure that FPE content are adapted to each patient and family’s needs. These findings agree with, and lend additional weight to, the existing evidence and guidelines which suggest that basic levels of systematic family involvement and FPE should be implemented as a standard approach in the treatment of persons with psychotic disorders. Findings from this study can guide future practice and pragmatic efforts to implement systematic family involvement in CMHCs.

## Data availability statement

The raw data supporting the conclusions of this article will be made available by the authors, without undue reservation.

## Ethics statement

The studies involving human participants were reviewed and approved by The Norwegian Regional Committee for Medical and Health Research (REC) Southeast. Registration number: 2018/128. The patients/participants provided their written informed consent to participate in this study.

## Author contributions

RP, KSH, and BW drafted the original research protocol, which this article is based on, and thus made significant contributions to the conception and design of the study. KMH, MR, LH, RP, and KSH provided the implementation support. KMH and MR performed the data collection. KMH, RP, MR, BW, LH, and KSH contributed to discussions on preliminary findings. KMH analysed and interpreted the data with major contributions from MR and RP, as well as contributions from LH. KMH wrote the first draft of this article, with major contributions from RP and MR, and further received substantial contributions from LH, IN, BW, and KSH. All the authors critically revised the article, gave their final approval before submission, and agreed to be accountable for all aspects of the work in ensuring that questions related to the accuracy or integrity of any part of the work are appropriately investigated and resolved.

## Funding

This study is funded by The Research Council of Norway (grant number 262863). This funding source had no role in the design of this study or during its execution, analyses, interpretation of the data, or decision to submit results.

## Conflict of interest

The authors declare that the research was conducted in the absence of any commercial or financial relationships that could be construed as a potential conflict of interest.

## Publisher’s note

All claims expressed in this article are solely those of the authors and do not necessarily represent those of their affiliated organizations, or those of the publisher, the editors and the reviewers. Any product that may be evaluated in this article, or claim that may be made by its manufacturer, is not guaranteed or endorsed by the publisher.

## Abbreviations

BFIS: basic family involvement and support; FPE, Family psychoeducation; IFIP, Implementation of Family Involvement for persons with Psychotic disorders; CMHCs, Community Mental Health Centres
