# Supplementary material for: “The most important thing is that those closest to you, understand you”: a nested qualitative study of persons with psychotic disorders’ experiences with family involvement
Source: Front Psychiatry. 2023 May 15;14:1138394. doi: 10.3389/fpsyt.2023.1138394 (PMC10225600; doi:10.3389/fpsyt.2023.1138394)
Supplement: Supplementary file 2 [file Data_Sheet_2.PDF]

The IFIP-study:
Patient interview guide

Introduction

This interview is part of the IFIP project, where community mental health centres (CMCHs) receive support to improve the collaboration between the user, their family and health personnel at the CMCH. The purpose of the project is that close family or other significant persons can support the user, and also get information and support themselves (e.g. by participating in a family group together, that the hospitals have appropriate information sharing routines, or that the user and relatives are taken care of by a family coordinator). The project is a collaboration between the University of Oslo (Centre for Medical Ethics), Akershus University Hospital HF (R&D unit of the Division of Mental Health), OsloMet (Labor Research Institute and Faculty of Health Sciences), The Early Intervention in Psychosis Advisory Unit for South East Norway (TIPS Sør-Øst) and the participating units, and it will end in 2022.

We have established a program of measures for family involvement in the CMCHs and we will evaluate whether these measures can improve the health and quality of life of both the users and their families. Your experiences are important because we need knowledge of how we can optimilise this collaboration.

In this interview, we are particularly interested in your experiences with family involvement. By “family involvement" we mean collaboration with the people who are important to you in your everyday life, and especially those who are closest to you. We would like you to use examples in the interview.

The interview will last for approximately one hour, and will be audio recorded and written down afterwards. Everything you say will be kept confidential and anonymised. Details that can identify you or others, such as names and locations, are removed when the interview is written. It is voluntary to participate, and you can withdraw at any time without explanation. It is possible that we will contact you again on a later occasion for supplementary information.

The participant fills in the consent form and returns page 3 to the researcher if they have not done this beforehand together with the therapist.

Start audio recording.

Overview of topics to be covered during the interviews

Family and family involvement

- Who is important to you in your everyday life? Has your therapist or anyone else at the CMCH been in contact with them?
- How have your closest relative(s) been involved in your treatment here at the CMCH? (*If necessary, exemplify: information, collaboration, support, seminar/course/group etc.)*
    - *If the participant does not mention family psychoeducation (FPE)/conversations:* Have you and your family participated in FPE and/or had one or more conversations about family involvement at the CMCH?
    - *If yes:* Who participated in these conversations, was the participant allowed to decide for himself who should participate, the number of participants, and performance?
    - *If no:* What is the reason(s) why your family has not been involved?
    - *(See Supporting questions Part 1 if necessary)*
    - Do you know if your relatives have had a conversation alone with your therapist or others at the CMCH? *If yes:* What do you think about that?

Experiences with- and significance of family involvement

- What has the family involvement meant to you?
    - How was it for you to participate in...? (*fill in what the participant has said about the involvement/collaboration). (See Supporting questions Part 2 if necessary)*
        - Was there anything you thought was good (about participating in...)?
        - Was there anything you found difficult (with participating in...)?
        - Do the therapists and your family listen to you? Are they interested in your opinions?
    - *If the participant has participated in FPE:* We know that some users experience that FPE has contributed to the process of recovery. What do you think about that? *If positive:* How has it been useful? *(See Supporting questions Part 3 if necessary).*
    - Was the form of collaboration important to your experience?
    - *If negative:* In what way do you feel that participation in FPE has been negative? *(See Supporting questions Part 4 if necessary)*

- What do you think the family involvement has meant to your family?
    - Do you perceive that your family/network has received support and help to understand what you are struggling with?
    - Have they been more helpful to you afterwards?

- For family involvement being helpful to you:
    - What do you want from your therapist?
    - What do you want from your closest relatives?

- Is there anything else you would like to share?

Supporting questions

Part 1 Reasons why the family has not been involved:

- Conflicts within the family
- Experiences of not being understood
- Worries and feelings of guilt in relation to that the family will be even more burdened if involved
- Negative thoughts about self-worth
- The illness is personal and private
- Shame and stigma
- The family gets too involved
- Care failure, other traumatic experiences (this should possibly not be thematised if the patient himself does not bring it up)

Part 2 After you and your family… (*fill in what the participant has said about the involvement/collaboration):*

Did you:

- Cope better socially?
- Feel that you had someone to turn to if you needed help?
- Get along well with your closest relatives?
- Experience increased or reduced mental health problems?
- Feel more or less satisfied with your life overall?
- Follow up on the treatment as agreed? (medication, appointments, other).

Have your relatives:

- Been more or less considerate towards you?
- Showed more or less understanding of your difficulties?
- Been more or less critical of you?
- Been more or less dissatisfied with what you do?
- Been hassling you more or less?
- Helped you in better ways?

With in the family:

- Is it easier to solve problems?
- Is it easier to communicate?

Did your therapists:

- Listen to what you consider is most important for your health situation?
- Take into account what is most important to you when choosing what to do next?

Part 3 FPE – What has been useful to you?

- Gaining knowledge and understanding about my own difficulties
- More openness/talk about psychological problems and symptoms
- Getting help to reduce stress
- Getting help to balance activities

- Getting support and help from the family
- Getting help to improve communication with in the family
- Getting help to solve everyday problems and challenges
- Getting help to prevent relapse and crisis

Part 4 FPE – Has family involvement been negative in any way?

- What happened that worsened your situation?
- How was this handled by the group leaders/therapists?
- Is there any particular topics you talked about that was difficult to you?
- How was it for you to participate in these meetings?
- What could have been done differently?
